# Design and development of an inflatable latex balloon to reduce rectal and bladder doses for patients undergoing high dose rate brachytherapy

**DOI:** 10.4103/0971-6203.48721

**Published:** 2009

**Authors:** P. Raghukumar, Raghu Ram K. Nair, Abi S. Aprem, Saju Bhasi, Suja Sisupal, V. Padmanbhan

**Affiliations:** Radiation Physics Division, Regional Cancer Centre, Trivandrum, Kerala-695 011, India; 1 M/s Hindustan Latex Ltd, Trivandrum, Kerala, India; 2 SUT Hospital, Pattom, Trivandrum, Kerala, India

**Keywords:** High dose rate brachytherapy, latex balloon packing, uterine cervix

## Abstract

Multiple fractions of High Dose Rate (HDR) brachytherapy along with external beam therapy is the common method of treatment for cancer of the uterine cervix. Urinary bladder and rectum are the organs at risk (OARs) that receive a significant dose during treatment. To reduce the dose to these organs, a majority of hospitals use vaginal gauze packing, as it is a simple, nontraumatic, and easy method. This article describes the design and development of an inflatable balloon that can be used along with the applicator as a substitute for gauze packing. The balloon has two parts-the bladder part (B-part) and the rectum part (R-part), both of them are independently inflatable. The selection of the material, its width, length, and thickness are described. A mould/former for making the balloon was designed. Polished steel was used as the mould. This was dipped in specially prepared natural rubber latex (NRL) solution several times; the layers were dried and stripped to get the balloon. The composition of NRL and the compounding recipe of the latex are also described. Physical tests like tensile strength, elongation at break, bursting volume, and radiation attenuation caused by the balloon, were checked. Biological tests for assessing type I and type IV allergies, like dermal irritation and skin irritation tests, were also done.

## Introduction

High Dose Rate (HDR) Brachytherapy using Ir-192 is now a standard procedure employed in the treatment of carcinoma of the uterine cervix. Multiple small dose fractions are given at weekly intervals using special vaginal and intrauterine applicators. Urinary bladder and rectum are the organs at risk (OARs) that receive a significant dose during treatment. In order to reduce the dose to these organs, methods like gauze packing in the vagina, rectal retractor, and shielded ovoids are used, which will move the organs out from the vicinity of the applicator containing the source.

A majority of hospitals use vaginal packing, as it is a simple, nontraumatic, and easy method. This can significantly reduce the dose to OARs provided the packing is adequate and uniform. Many studies[1,2] suggest that inadequate vaginal packing can increase the dose to OARs and reduce the therapeutic ratio. Since the treatment involves multiple fractions, for every fraction the gauze must be uniformly packed, failing which, following dose computation, either repacking will have to be done or a compromise to the prescription dose will have to be made. To overcome the above problem, a special latex balloon device was designed to replace the gauze, as packing material. The design and development of the latex balloon is described in this article.

## Materials and Methods

The balloon consists of two parts - one on the rectal side and the other on the bladder side, joined together. The design criteria adopted for the development of the inflatable balloon includes the selection of (1) the material, (2) the required width, (3) the length, and (4) thickness.

The selection of the material for the balloon should satisfy the criteria regarding biocompatibility. The material selected should not cause any allergy or toxicity to the patient. The physical form of the material should not produce any trauma to the patient. It should be free from any type of pointed edges to exclude any injury to the vagina. The material should not macerate like the gauze pack during the procedure. It should be soft and should not break or burst on expansion. The introduction of the balloon should not alter the intensity distributions emitted by the source. It means that the attenuation of the radiation beam by this device in situ should be negligibly small so that the lateral throw off dose should not be affected. Since natural rubber latex (NRL) has proved its role as a safe material that can be used in contact with biological tissue, the present study has used double-centrifuged NRL for making the balloon. Table 1 shows the composition of NRL.[3] Biological and physical tests were done on this material, and the tensile strength and elongation at break were measured.

**Table 1:** Composition of natural rubber latex

*Components*	*Amount (%)*
Rubber	30-40
Protein	1-1.5
Resin	1-2.5
Sugar	1
Ash	<1
Water	55-60

The width of the balloon was decided by the standard setting of the applicator (Standard Gynecological Applicator from Nucletron India Pvt. Ltd.) with an Intra Uterine (IU) tube and two medium ovoids. The anteroposterior (AP) projection of the standard applicator with IU and ovoids in locked position was taken onto a paper and distance from the left-most surface of the left ovoid to the right-most surface of the right ovoid was assumed as the maximum width of the balloon (=5.5cm). On expansion, the surface area of the balloon that touched the ovoids would increase and provide enough seating space for the ovoids. The diameter of the flange was 2cm. There was no need to put the same width of 5.5cm at the joining part of the balloon, because the part of the balloon in touch with the flange should not expand to displace the cervix away from the applicator. Hence, the width at the joining part was made equal to the diameter of the flange (=2cm).

The length of the vagina varies from patient to patient. The balloon is placed in the vagina along with the applicator. Its length must not be so long that it projects out of the vagina. After a series of measurements in patients and considering the geometry of the applicator, a length of 7.5cm was selected while making the balloon.

**Figure 1:**
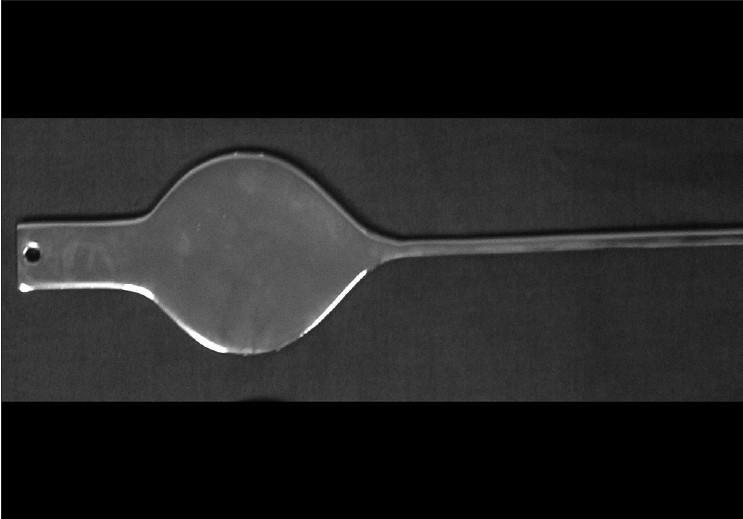
Stainless steel former (mould) used for making the balloon

**Figure 2:**
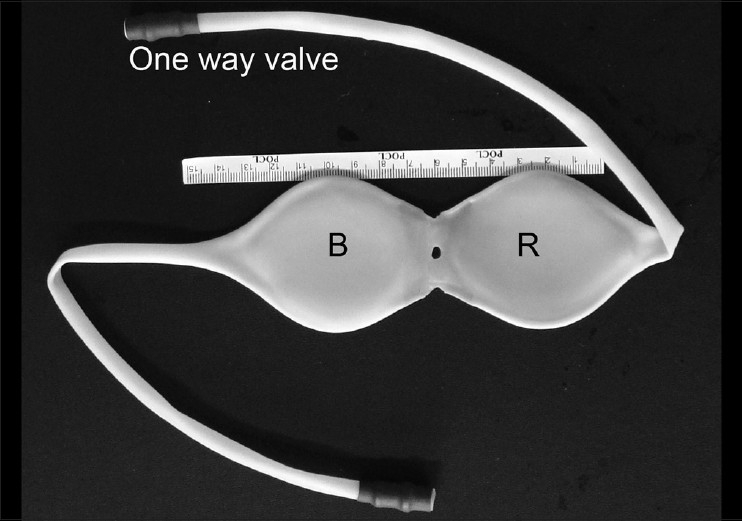
The balloon showing the B and R parts and one-way valves at the tail ends

Using these dimensions (width = 5.5cm, length 7.5cm) a 0.5cm thick mould in stainless steel was designed, without any pointed edge, as shown in Figure 1. This mould was dipped several times in the specially prepared double-centrifuged NRL (same material that is used for making condoms) for achieving the required thickness. The composition of this preparation is shown in Table 2.[4] As the latex dried, it was stripped off from the mould to form a balloon. Two such balloons named as B (bladder) and R (rectum) were joined together using latex adhesive. A hole was made at the joining part to negotiate the IU tube. One-way valves were attached to the tail ends of the balloons, which would prevent the escape of fluid pushed in. The final product obtained is shown in Figure 2.

**Table 2:** Compounding recipe of the material used

*Ingredients*	*Parts/wt(100 g rubber)*
60% NR Latex	166
Con.NH3	1.2
Sulfur	1.5
Zinc oxide	0.9
Phenolic antioxidant	0.5
Dispersing agents	0.105
Casein	1.0
Potassium oleate	0.6
Primary accelerator	0.5
Secondary accelerator	0.2

The thickness of the latex sheet for making the balloon was found by checking the behavior of the balloon inside the patient, on expansion. At the onset of the trial, a balloon having the same thickness on both sides was used, on the assumption that the part in contact with the applicator would expand into the space between the applicators and hold it in place. However, on use it was found that it expanded differently and was not consistent between fractions. Therefore, it was decided to reduce the expansion of the region that was in contact with the applicator to a minimum, compared to the other side. This was achieved by increasing the thickness of that region (the region in contact with the applicator). Hence, each side (bladder and rectal) of the balloon had a different thickness. The average thickness of the product in use for the thin part was 0.50 mm and for the thicker part was 1.7 mm. Again, on use it was found that the lower portion of the vagina required less expansion as it was away from the source. In order to achieve this, the lower part of the thin side was made thick.

## Bursting Volume

The bursting volume of the B and R parts of the balloon was measured by pushing water into the balloon using a graduated syringe.

### Tensile strength and elongation at break

Tensile strength is the maximum tensile stress reached when stretching a flat dumbbell shaped specimen to its breaking point. By convention, the force required is expressed as the force per unit area of the original cross section. Elongation at break or ultimate elongation at the time of rupture is expressed as a percentage of the original distance marked on the test piece before applying force. Both tests were done before and after aging of the latex specimen. Aging was done by keeping the specimen in an air oven at 70°C for four days.

The parameters were measured on a dumb-bell shaped test piece as per American Society for Testing and Materials standard (ASTM D412). The thickness of the narrow portion of the specimen was measured using a dual gauge. The ends of the test specimen were fixed to the Universal Testing Machine (Make: INSTRON, Model: 4400) to measure these parameters.

### Attenuation of radiation beam

The attenuation caused by the balloon was found by interposing it between the chamber and Ir-192 source. For this measurement, the same set-up was used as that for the in-air measurement of source strength, using the Nucletron's Jig. The position of maximum sensitivity was found by dwelling the source at different positions within channel 1. Without disturbing the experimental set-up, latex sheets of known thicknesses were introduced inbetween the chamber and source, and the corresponding readings (R_t_) were noted by dwelling the source at the position of maximum sensitivity. Two samples of balloons were randomly selected. The B and R parts of the balloons were introduced separately and readings were noted. The attenuation caused by the B and R parts of the balloon was found by dividing the readings (R_t_) with the readings obtained using zero thickness (R_o_).

## Results

The results obtained after measuring the tensile strength are shown in Table 3. The tensile strength of the NRL material, measured in Mega Pascal (MPa), before and after the thermal aging process should fall in the range of 17 to 35 MPa.[5] In the present study it varied from 20.37 to 22.97 MPa.

**Table 3:** The tensile strength and elongation at break for latex material

*Sample no.*	*Before aging*		*After aging*	
		
	*Tensile strength (MPa)*	*Elongation (%)*	*Tensile strength (MPa)*	*Elongation (%)*
1	21.74	773.15	20.57	729.64
2	22.41	805.69	20.67	762.97
3	22.97	784.74	20.37	739.73

The bursting volume was measured for the B and R parts of the balloon.

Both parts could withstand volumes above 800cc of water/air without bursting. In a clinical situation, not more than 50cc will be required in each part.

The radiation attenuation produced by the balloon was measured. This was less than 1% for each part, for the samples tested [Table 4].

**Table 4:** Attenuation of NRL sheets and balloon samples

*Thickness of latex sheets (mm)*	*Readings (pC) obtained at the position of maximum sensitivity for 60 sec*			*R_t_/R_o_*
		
	*1*	*2*	*Mean*	
0.00	537.0	537.0	537.0	1.0000
0.95	536.5	536.0	536.3	0.9986
1.83	534.5	534.5	534.5	0.9953
2.72	532.5	532.0	532.3	0.9912
3.54	531.0	531.0	531.0	0.9888
4.31	529.0	529.5	529.3	0.9856
5.16	527.0	527.0	527.0	0.9814
Sample1-R	533.5	533.5	533.5	0.9935
Sample1-B	533.0	533.0	533.0	0.9926
Sample2-R	533.5	533.5	533.5	0.9935
Sample2-B	534.0	534.0	534.0	0.9944

## Biological Studies

Any contact with articles manufactured from natural rubber may produce dermatitic conditions (e.g., allergic contact eczema) in certain individuals. These (Type IV) skin allergies are caused not by the rubber itself, but by additives such as accelerators and antioxidants. Another kind of allergy (Type I) is caused by a protein, native to Hevea latex. Type I allergies are sometimes referred to as ‘immediate allergies’ because the response on exposure to the allergen is fast compared with the time scale of the reactions in Type IV allergies.

Dermal Irritation and Skin Sensitization Tests were done on the balloon at the Toxicology Division, Vimta Laboratories Ltd., Hyderabad. The study included the following tests described by the International Organization for Standardization (ISO)[6] and the Organization for Economic Co-operation and Development (OECD)[7]

1. Primary Skin Irritation tests in Albino rabbits
2. Irritation to Vaginal Mucosa in Female Albino Guinea pigs
3. Skin Sensitization Test in Albino Guinea pigs

All these tests produced no adverse reactions in the animals.

## Conclusion

Natural rubber latex was used for making a two-compartmental balloon device that could be used as a substitute for vaginal gauze packing. Factors like applicator size, length of the vagina, etc., were considered for selecting the length, width, and thickness of the balloon. A mould was fabricated using stainless steel for making the balloon. The balloon was tested for its physical and biological qualities.

It is found that the design of the balloon satisfies all the requirements of a packing device that can be substituted for gauze and safely used in intracavitary high dose rate brachytherapy. The balloon is disposable and is meant for single use only. A clinical evaluation of the balloon has been done and will be reported.

The design is pending patent (No.857/CHE/2006A).
